# Magnetic resonance imaging of sellar and juxtasellar abnormalities: atypical findings of common diseases and typical findings of rare diseases

**DOI:** 10.1590/0100-3984.2016.0039

**Published:** 2018

**Authors:** Denislene da Silva Eduardo, Suyane Benevides Franco, José Daniel Vieira de Castro

**Affiliations:** 1 Médica Radiologista da São Carlos Imagem, Fortaleza, CE, Brasil.; 2 Médica Residente de Radiologia e Diagnóstico por Imagem, Universidade Federal do Ceará (UFC), Fortaleza, CE, Brasil.; 3 Doutor, Médico Neurorradiologista, Professor Associado de Radiologia do Departamento de Medicina Clínica da Faculdade de Medicina da Universidade Federal do Ceará (UFC), Fortaleza, CE, Brasil.

**Keywords:** Sella turcica, Magnetic resonance imaging, Pituitary gland

## Abstract

The sellar/juxtasellar region comprises the bone component of the sella turcica, pituitary gland, cavernous sinus, and suprasellar cistern. Abnormalities in this region can be attributed to underproduction or overproduction of hormones or to the neurological signs and symptoms resulting from the compression of adjacent structures. Magnetic resonance imaging (MRI) is currently the imaging method of choice, having supplanted computed tomography. The aim of this study was to demonstrate the common and uncommon imaging aspects of sellar and juxtasellar changes, which could facilitate the differential diagnosis. We retrospectively evaluated the MRI scans of 70 patients with sellar/juxtasellar abnormalities from didactic files, and report those with more unusual changes, where MRI played an important role in diagnosis. All cases were confirmed histologically or clinical laboratory.

## INTRODUCTION

The sellar region, albeit small, encompasses a number of important structures, including the bone component of the sella turcica, as well as the pituitary gland, cavernous sinus, and suprasellar cistern. Abnormalities in this region can be attributed to the underproduction or overproduction of hormones or to the neurological signs and symptoms resulting from the compression of adjacent structures. Virtually any of those factors can lead to disease, ranging from the innocuous to the severe^(^^1,2^^)^. Magnetic resonance imaging (MRI) is currently the method of choice, having supplanted computed tomography (CT), for evaluation of the sellar/juxtasellar region^(^^3^^)^.

Although pituitary adenomas are the most common lesions in the sellar/juxtasellar region, lesions originating in other structures can also affect the region, complicating the diagnosis. We retrospectively evaluated 70 cases of sellar/juxtasellar abnormalities, confirmed by histopathology or on the basis of clinical and biochemical findings, in order to describe the common and unusual aspects of these alterations that aid in the differential diagnosis.

## ADENOMA

Pituitary adenomas represent the most common sellar lesions. They are classified, by size, as macroadenomas (≥ 10 mm) or microadenomas (< 10 mm) and, by hormone production, as secreting or nonfunctioning^(^^4-6^^)^.

**Figure 1:**
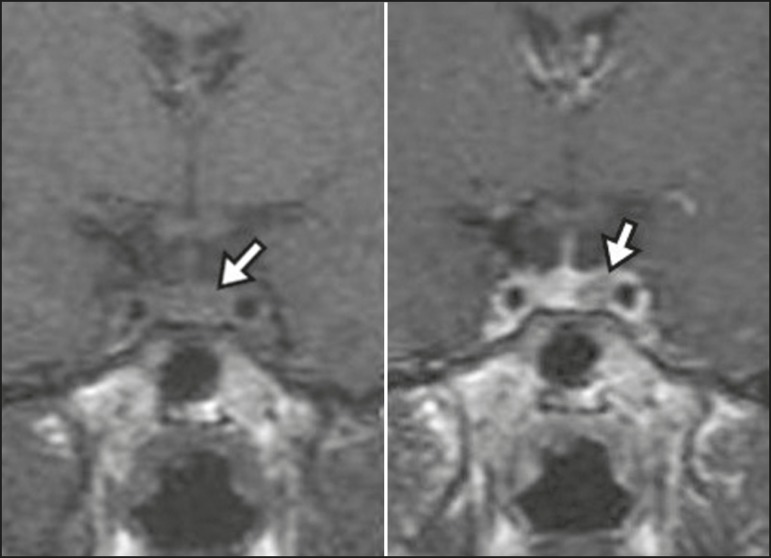
Dynamic contrast-enhanced coronal T1-weighted MRI sequences showing a microadenoma in the left portion of the adenohypophysis (arrows). Note that contrast enhancement was slower in the microadenoma than in the normal pituitary parenchyma.

On MRI, adenomas typically present hypointense signals in T1-weighted sequences and signals of variable intensity in T2-weighted sequences. One important aspect is that, due to those hypointense signals, the majority of adenomas can be detected in T1-weighted sequences, which makes such sequences the most important of the MRI protocol for the evaluation of this alteration, given that it allows the diagnosis even without contrast administration. Another important aspect is that most adenomas present post-contrast enhancement slower than does the normal parenchyma (Figure 1), making it important to use dynamic contrast-enhanced MRI sequences for the detection of microadenomas^(^^7-9^^)^.

**Figure 2:**
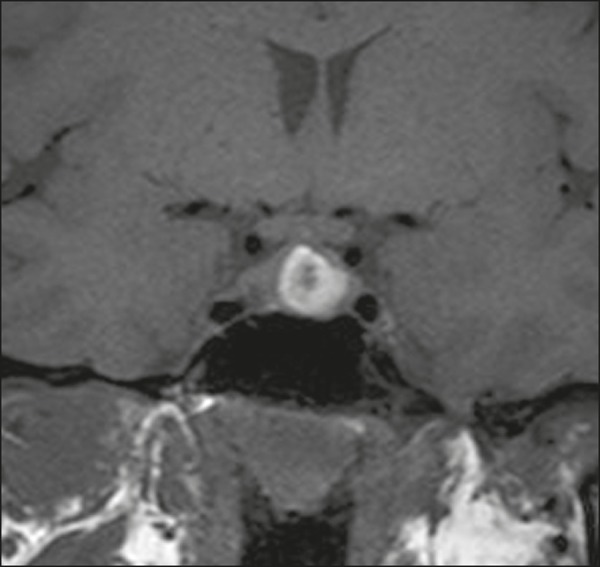
Non-contrast-enhanced coronal T1-weighted MRI sequence showing a prolactinoma with heterogeneous signal intensity that was predominantly hyperintense after therapeutic management, due to intralesional hemorrhage.

Among secreting adenomas, the most common are prolactin producers (prolactinomas). In most cases, prolactinomas can be treated exclusively with dopaminergic agonists, although such treatment can result in alterations to the imaging aspects (Figure 2), which must be recognized by the radiologist^(^^6^^)^.

**Figure 3:**
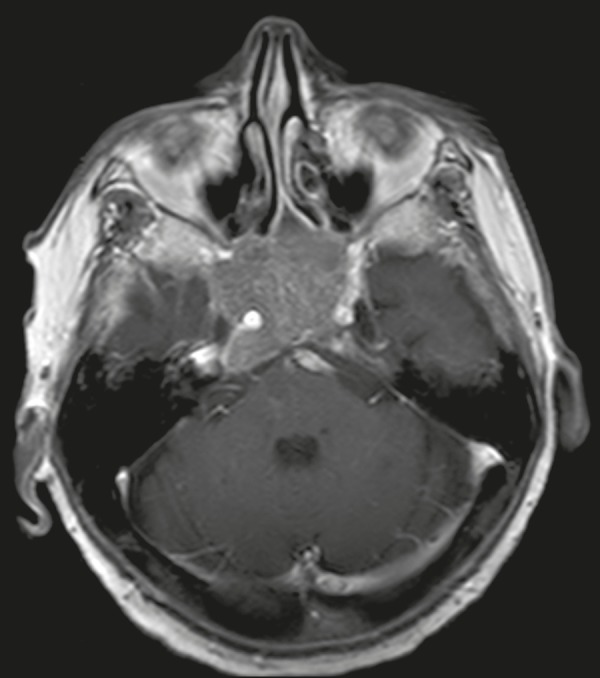
Contrast-enhanced axial T1-weighted MRI sequence showing a macroadenoma invading the cavernous sinus, although not altering the caliber of the left carotid artery.

Macroadenomas sometimes extend beyond the boundaries of the sellar region, invading the cavernous sinus, sphenoid sinus, or clivus, as well as compressing the optic chiasm and enveloping the internal carotid artery (Figure 3). On MRI, invasion of the cavernous sinus is defined as a situation in which at least two-thirds of the circumference of the cavernous segment of the internal carotid artery is encompassed by the lesion. Therefore, it is occasionally necessary to make the differential diagnosis with other lesions that can occur in this region, such as meningiomas and even aneurysms^(^^4,5^^)^. Large adenomas are usually heterogeneous, containing cystic areas resulting from cystic degeneration or necrosis, and can occasionally develop infarction or hemorrhage, due to poor vascular supply^(^^3,8^^)^.

## CRANIOPHARYNGIOMA

Craniopharyngiomas are slow-growing epithelial neoplasms that originate from the remnant of the craniopharyngeal duct and account for 3-5% of intracranial neoplasms. Their incidence shows two peaks, the first occurring between 10 and 14 years of age and the second between the fourth and sixth decade of life. Although craniopharyngiomas are suprasellar in origin, approximately 50% extend into the sellar region. The typical appearance includes solid-cystic components and calcifications^(^^3,5,9^^)^.

**Figure 4:**
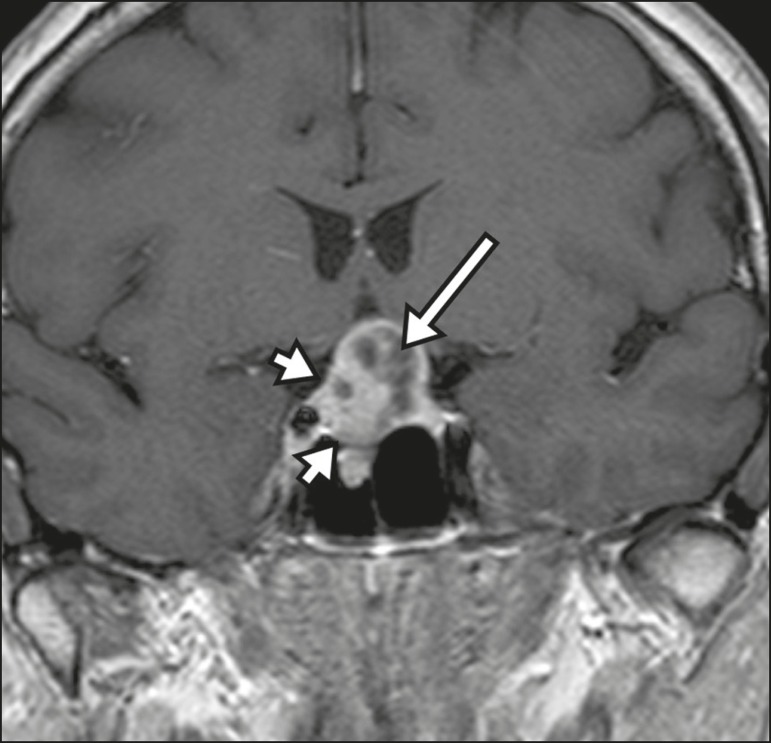
Adamantinomatous craniopharyngioma. Contrast-enhanced MRI scan showing a cystic component (long arrow) and a solid component (short arrow), both of which show some degree of contrast enhancement.

The classic, adamantinomatous, type of craniopharyngioma has a cystic appearance and contains heterogeneous nodules. The least common, squamous papillary, type has a predominant solid component. In T2-weighted MRI sequences, the cystic component shows a hyperintense signal, whereas the solid components show heterogeneous signals. After contrast administration, the solid portions show intense heterogeneous enhancement and there is enhancement of the cystic walls (Figure 4)^(^^3,8^^)^.

**Figure 5:**
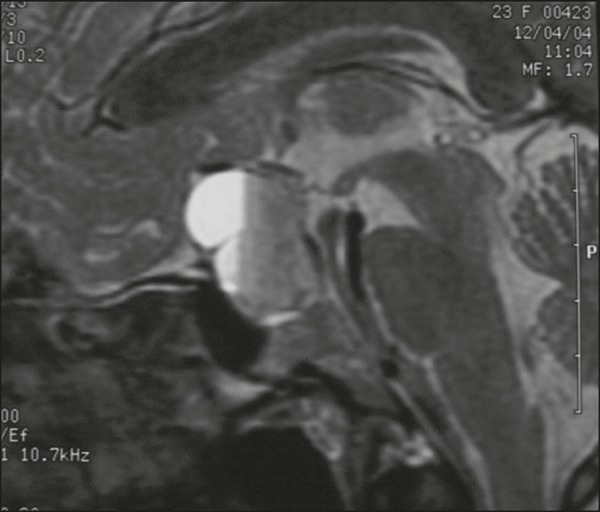
Macroadenoma with pituitary apoplexy. Sagittal T2-weighted MRI sequence showing an expansile lesion extending into the suprasellar region, with a fluid-fluid level and signs of intralesional hemorrhage.

**Figure 6:**
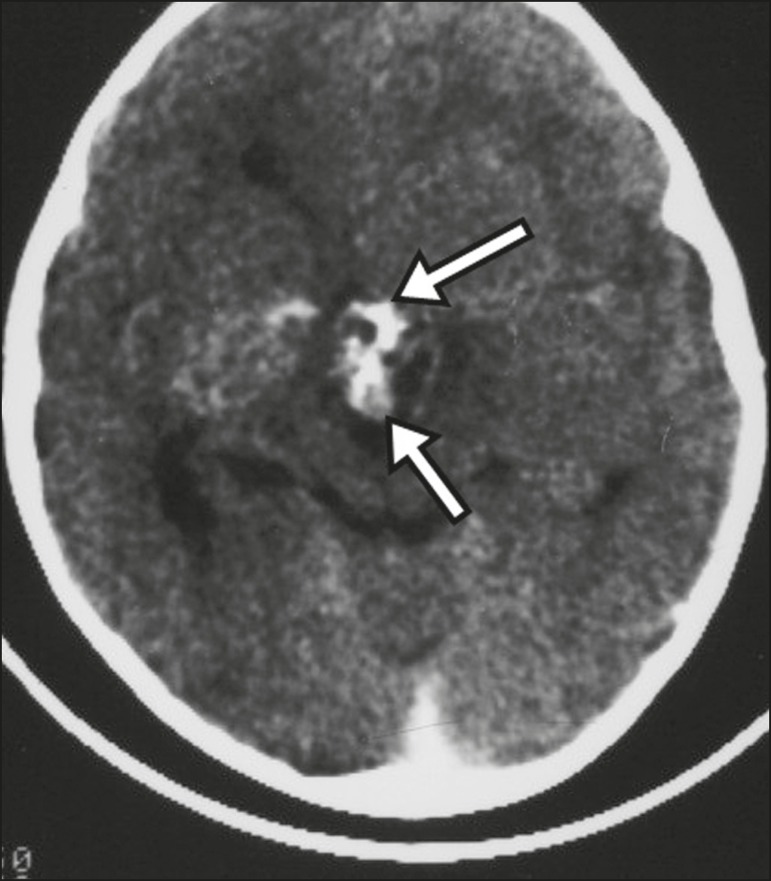
Craniopharyngioma. Non-contrast-enhanced CT scan showing calcifications (arrows) within the lesion, which constitute an important finding for diagnostic imaging.

Although macroadenomas with pituitary apoplexy (Figure 5) and Rathke's cleft cysts can have aspects quite similar to those of craniopharyngiomas, an important distinguishing aspect of craniopharyngiomas is the presence of calcifications. Therefore, when the MRI findings are inconclusive for calcifications, non-contrast-enhanced CT should be performed in order to confirm their presence and corroborate the diagnosis (Figure 6).

## RATHKE'S CLEFT CYSTS

**Figure 7:**
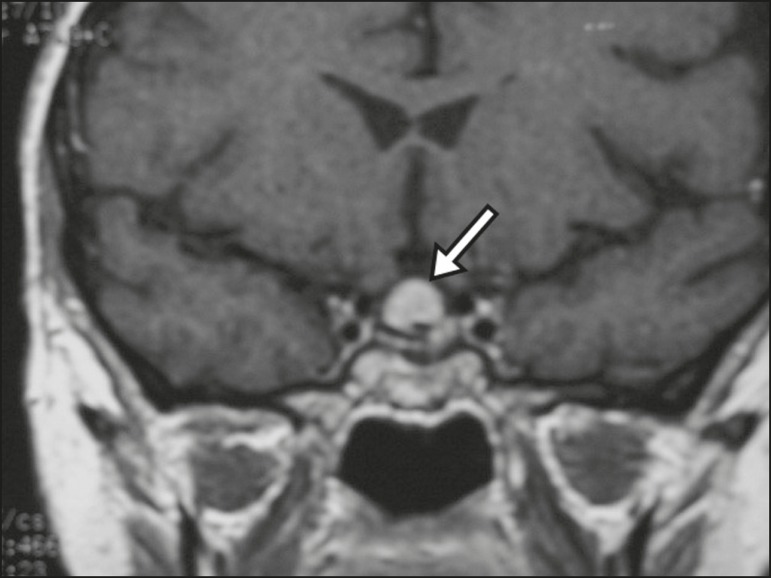
Rathke’s cleft cyst. Coronal T2-weighted MRI sequence showing an expansile cystic lesion extending into the suprasellar region, with regular contours and thin walls (arrow).

Rathke's cleft cysts are benign, often asymptomatic, lesions of the sellar region, most often being intrasellar. On MRI, they usually show a hyperintense signal in T2-weighted sequences, whereas they can show hyperintense or hypointense signals on T1-weighted sequences, depending on their protein content (Figure 7). The differential diagnosis of Rathke's cleft cysts always includes craniopharyngioma. The absence of calcifications favors the diagnosis of a Rathke's cleft cyst^(^^6^^)^.

## MENINGIOMA

**Figure 8:**
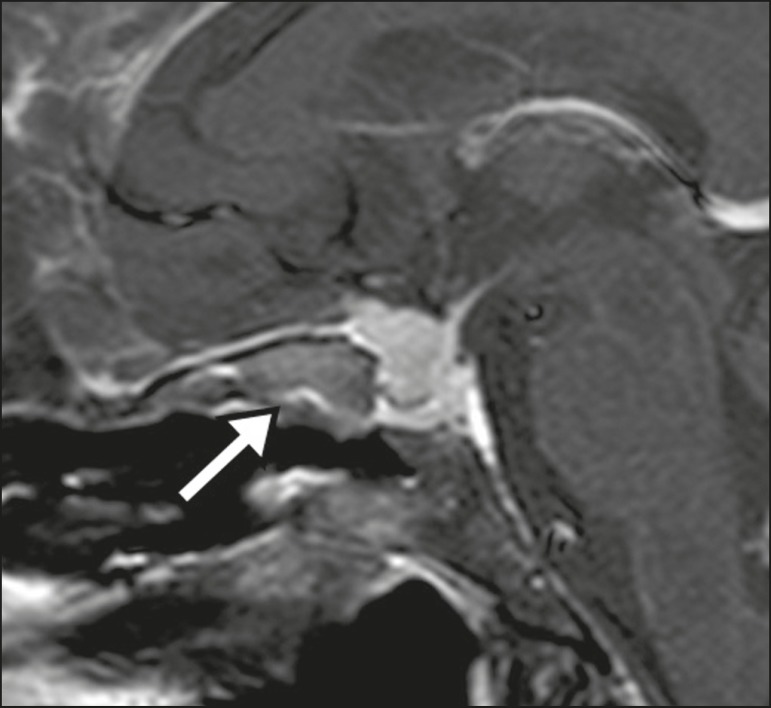
Suprasellar meningioma in a sagittal T1-weighted MRI sequence. Note the hyperostosis of the sphenoid bone (arrow) and the distinct separation from the sella turcica by the sellar diaphragm, findings that support the diagnostic hypothesis of meningioma.

**Figure 9:**
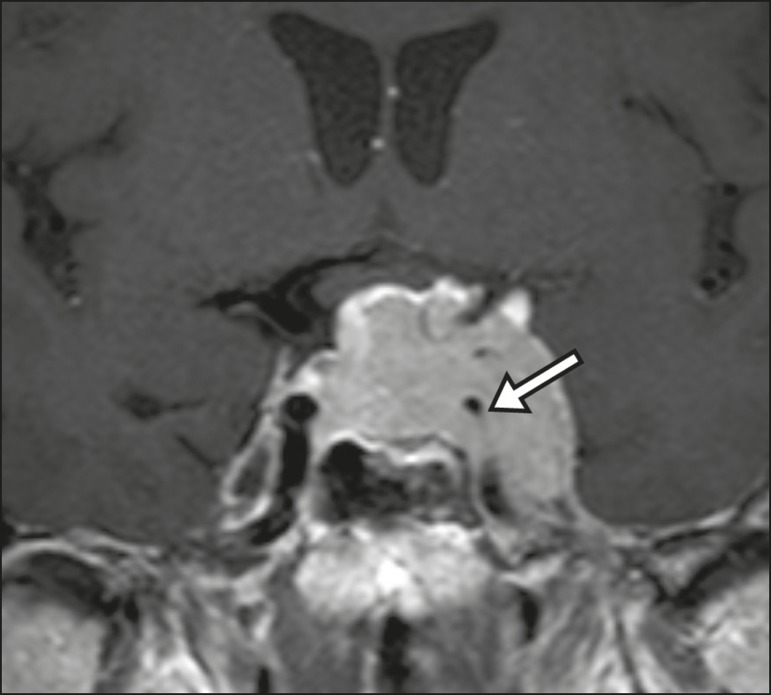
Large meningioma invading the left cavernous sinus. Gadoliniumcontrast- enhanced coronal T1-weighted MRI sequence showing homogeneous enhancement and circumferential involvement of the left cavernous sinus. Note the reduction in the caliber of the lumen of the cavernous portion of the left internal carotid artery (arrow), a finding that is highly suggestive of meningioma.

Sellar meningiomas account for 20-30% of all intracranial meningiomas. On MRI, sellar meningiomas show an isointense signal in T1-weighted sequences and an isointense or hyperintense in T2-weighted sequences, as well as early enhancement, usually accompanied by the dural tail sign (Figure 8). When they invade the cavernous sinus, they tend constrict the carotid artery (Figure 9), which rarely occurs in cases of adenoma. The presentation of a sellar meningiomas can also include calcifications and hyperostosis^(^^5-8^^)^.

## ANEURYSM

**Figure 10:**
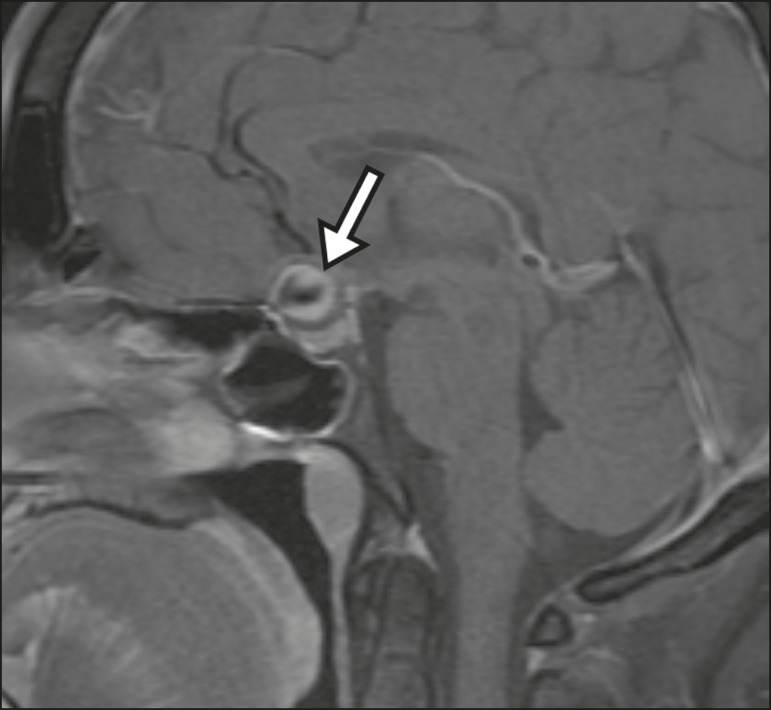
Intrasellar aneurysm identified by a flow void (arrow), due to the high velocity flow, in a T1-weighted MRI sequence.

**Figure 11:**
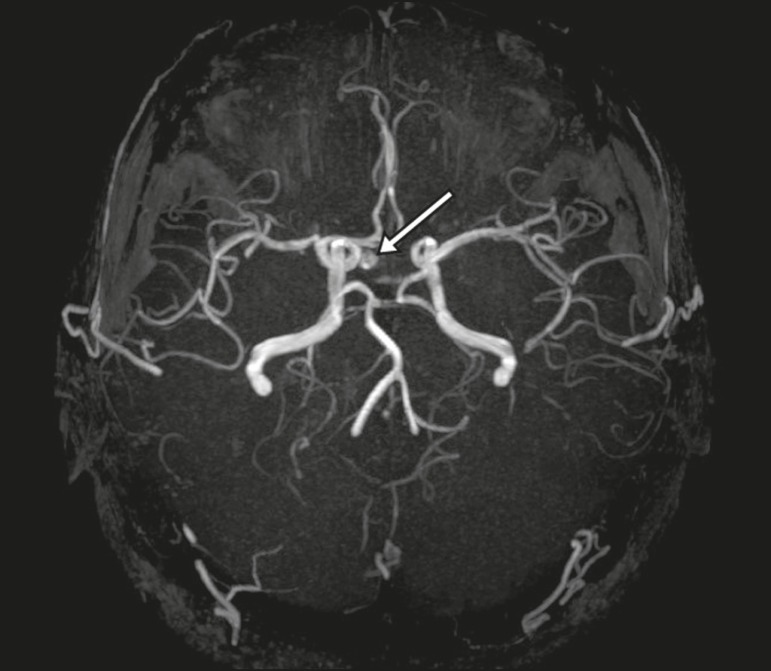
Aneurysm (arrow) of the cavernous portion of the right internal carotid artery, protruding into the sella turcica. Although the presence of partial thrombosis generated diagnostic confusion with hemorrhagic adenoma, MR angiography clarified the diagnosis.

Aneurysms of the sellar region typically originate from the cavernous or supraclinoid portion of the internal carotid artery, accounting for up to 10% of all cerebral aneurysms. Their diagnosis is made more easily with MRI than with CT, because the former can reveal a flow void, due to the rapid luminal flow, and heterogeneous signal intensity in areas with slow, turbulent flow (Figure 10). However, thrombosed aneurysms can occasionally cause diagnostic difficulties, as described in Figure 11^(^^3,5^^)^.

## HYPOTHALAMIC HAMARTOMA

**Figure 12:**
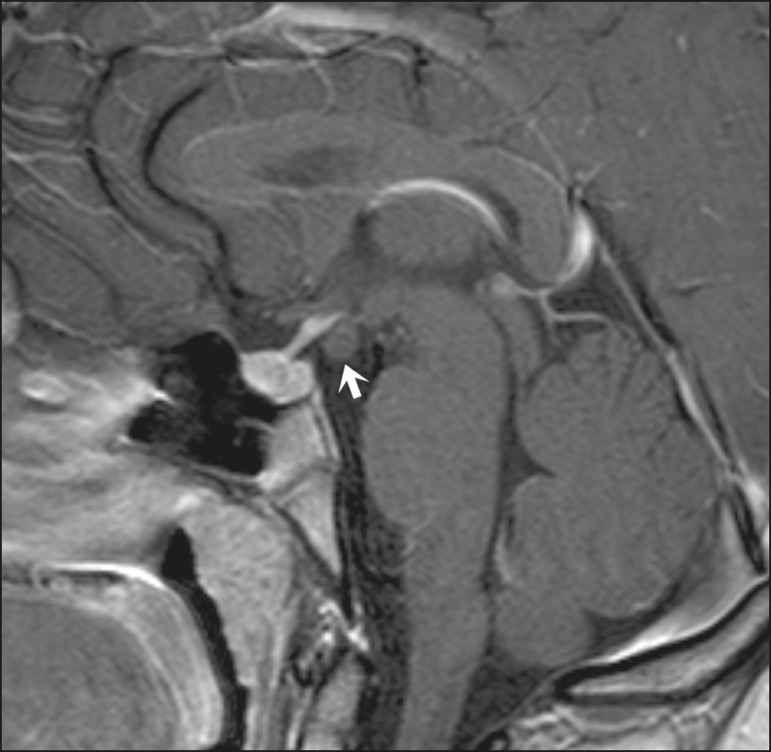
Contrast-enhanced sagittal T1-weighted MRI sequence showing a parahypothalamic hamartoma, between the infundibular stalk and the mammillary bodies (arrow), in a child with precocious puberty.

**Figure 13:**
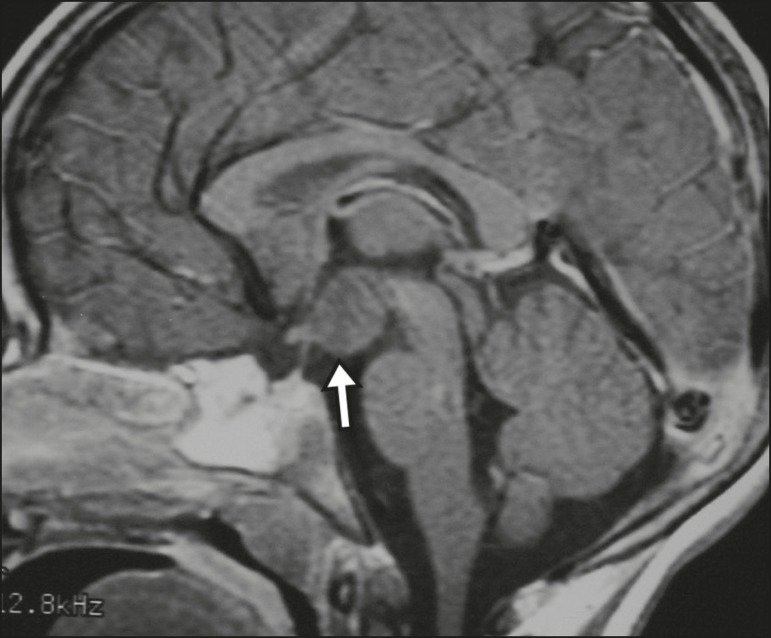
Sagittal T1-weighted MRI sequence showing an intrahypothalamic hamartoma (arrow) in a child with gelastic seizures.

Hypothalamic hamartomas consist of ectopic foci of neural tissue (gray matter), typically located in the tuber cinereum and mammillary bodies. They typically manifest as an increase in the size of the tuber cinereum. On MRI, hypothalamic hamartomas present signals that are, in comparison with that of the gray matter, isointense in T1-weighted MRI sequences (Figure 12) and isointense or hyperintense, without contrast enhancement or calcifications, in T2-weighted sequences. They can be parahypothalamic or intrahypothalamic (Figure 13), the latter more often being associated (clinically) with epilepsy, including gelastic seizures, whereas the former are more often associated with precocious puberty. The stability of hypothalamic hamartomas over time facilitates the differential diagnosis with other lesions occurring in the same region, such as gliomas^(^^3,5,6,9^^)^.

## HEMANGIOMA

**Figure 14:**
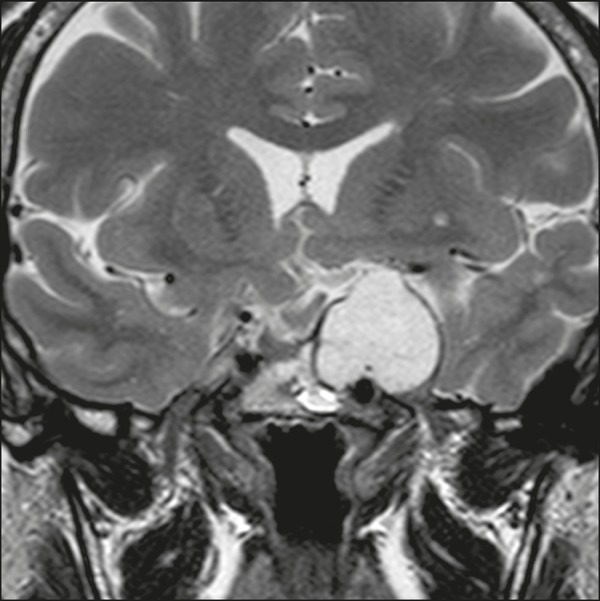
Hemangioma. Coronal T2-weighted MRI sequence showing a left juxtasellar lesion with a markedly hyperintense, homogeneous signal.

**Figure 15:**
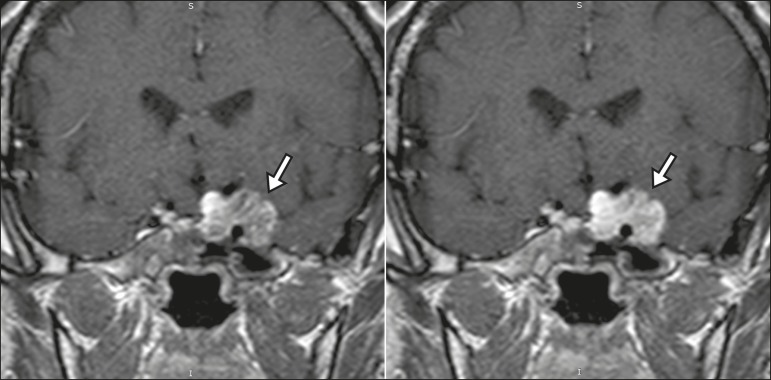
Hemangioma. Dynamic contrast-enhanced coronal T1-weighted MRI sequences showing initial peripheral contrast enhancement with subsequent centripetal filling.

Hemangiomas constitute vascular malformations found in various organ systems, including the central nervous system. When extracerebral, they can originate from the cavernous sinus or from the adjacent tissues. Like hepatic hemangiomas, hemangiomas in the sellar region manifest on MRI as well-defined masses with hypointense or isointense signals in T1-weighted sequences and markedly hyperintense signals in T2-weighted sequences (Figure 14), initially with peripheral contrast enhancement, centripetal filling leading to late homogeneous enhancement. Therefore, dynamic contrast-enhanced MRI (Figure 15) is essential for the accurate characterization of the lesion^(^^10^^)^.

## HYPOPHYSITIS

**Figure 16:**
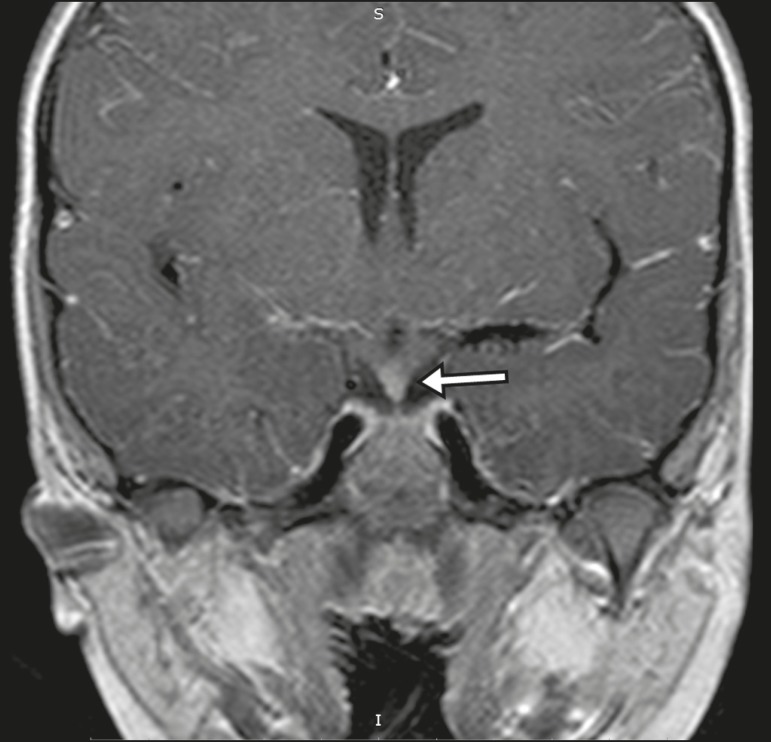
Non-contrast-enhanced coronal T1-weighted MRI sequence, showing thickening of the pituitary stalk and enhancement (arrow), in a patient with Langerhans cell histiocytosis.

Inflammation of the pituitary gland, or hypophysitis, comprises a complex group of diseases, with two main histological forms: lymphocytic (the most common, autoimmune, form); and granulomatous (secondary to infection, sarcoidosis, or Langerhans cell histiocytosis). Because it is practically impossible to distinguish between the two forms on the basis of the radiological findings, the clinical history has great value in the differential diagnosis. On MRI, hypophysitis presents as thickening of the pituitary gland in combination with intense contrast enhancement, as shown in Figure 16^(^^1,5,11,12^^)^.

## ECTOPIC NEUROHYPOPHYSIS

**Figure 17:**
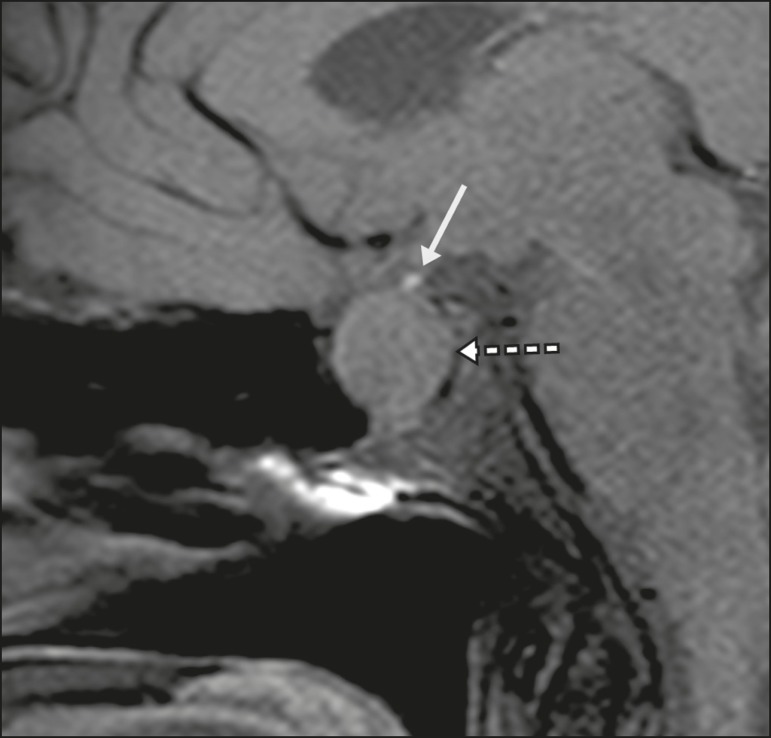
Sagittal T1-weighted MRI sequence with fat saturation, showing an ectopic neurohypophysis (solid arrow), secondary to a macroadenoma (dashed arrow).

**Figure 18:**
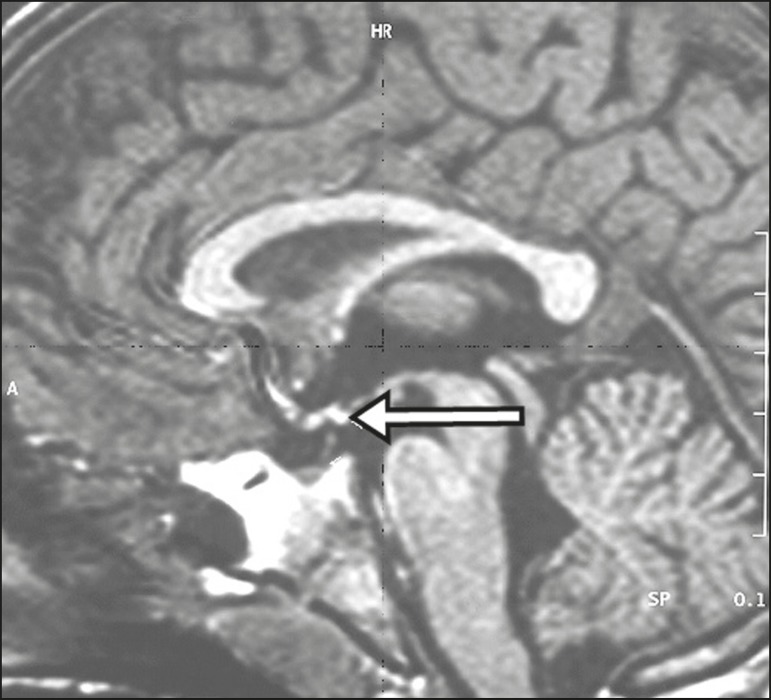
Sagittal T1-weighted MRI sequence showing an ectopic neurohypophysis (arrow) in a patient with idiopathic growth hormone deficiency.

Normally, the neurohypophysis is located within the sella turcica, posterior to the adenohypophysis. It consists of the terminal axons of neurons projected from the hypothalamus, differentiated to store oxytocin and the antidiuretic hormone. Ectopic neurohypophysis occurs in three situations: when there is compression of the pituitary stalk by an expansile lesion (Figure 17); when a trauma has injured the pituitary stalk; and when there is a congenital anomaly (Figure 18). The last situation is associated with idiopathic growth hormone deficiency^(^^11,13,14^^)^.

## CONCLUSION

The great number of lesions that can affect the sellar/juxtasellar region requires that radiologists not only possess knowledge of the anatomy and the contents of this region but also familiarize themselves with the various possible aspects of such lesions. In most cases, the application of such knowledge can lead to an accurate etiological diagnosis.
